# The Audiphone

**Published:** 1880-04

**Authors:** 


### THE AUDIPHONE.

Two instruments have recently appeared for which their originators claim effects that are not substantiated in the tests made of them. First, we have the audiphone, the inventor of which claims to enable all manner of deaf people to hear conversation through the instrumentality of his contrivance. Believing that *some* forms of deafness might be benefitted by this instrument, we secured one for experiment. The results of our investigations we give for the benefit of many deaf persons who have addressed inquiries to us relative to the usefulness of the instrument.

The audiphone consists of a piece of thin, vulcanized rubber, about a foot square, with rounded corners and a solid handle upon the center of one edge, resembling, very much, a Japanese fan. Four holes are made upon the margin opposite the handle, two near the outer edge, and two more at equal distances between, on the same line. Through these holes are placed two silken cords, their four free ends being tied at a point opposite the center of the fan, from which point a single cord runs to the handle, where it is secured in such a manner as to draw the top of the fan toward the handle, thus causing it to become concave. This is the audiphone. By taking a thin piece of dry cedar, or even pine, and gluing a handle to it, stringing it in the manner described, an equally-good instrument is obtained, at a cost that need not exceed twenty-five cents. The price asked for the advertised instrument is exorbitant and should not be paid by any one, particularly as it is extremely doubtful whether any benefit will be derived from it by the purchaser.

When the cords of the audiphone are drawn taut, bending the disk in the manner above described, its upper edge is placed in contact with the incisor teeth, the covex side being held outward. If your teeth are false ones, you cannot use the concern, solid teeth firmly imbedded in the jaw, being the requisites for the vibrations to be conducted to the auditory nerve. The instrument must be held firmly against the teeth; then, if the auditory nerve is sound, not in the least impaired through scarlet or typhoid fevers, cerebro-spinal and other brain ailments, syphilis, old age and many other causes that could be mentioned, *perhaps* you may hear a voice, when loudly exerted against the diaphragm. But, to get the best results, you must be almost entirely deaf. We have found that very little or no aid at all (most frequently the latter) is afforded the partially deaf, or those who can hear very loud conversation unaided by instruments of any kind.

The results obtained from the instrument, as far as we have applied it, might be summed up about as follows : It is of no earthly use to any one, save deaf-mutes, and they are only enabled to *hear sounds.*

The other instrument, called the dentaphone, is constructed upon the same principles, but has a vibrating disk like a telephone, from which runs a cord to a hard rubber mouthpiece that is held between the teeth, and is about of the same value. Our advice is not to invest any money in either. Construct a wooden one, when, if any aid to the hearing is obtained, a more costly one could be employed, if desired.

These instruments are constructed upon the well-known law that sound waves are communicated, through the air, to surrounding bodies, causing them to vibrate in unison, with varying degrees of intensity, according to the resonance of the body acted upon. It is also a well ascertained fact that vibrations of sound can be transmitted in longitudinal directions through resonant substances like wood. This has been illustrated in receiving the sound of taps through a long log, or by placing the ear to the end of a wall and distinctly hearing the raps applied at the opposite end, however distant. Almost, if not quite as good results as are experienced from the use of the audiphone may be obtained by applying a dry pine stick, [two feet long and an inch in diameter] to the teeth, or any of the bones of the skull, of the deaf person, while the opposite end is held in the same position by the person conversing. Sounds can be heard by the deaf, or partially deaf—such as the ticking of a watch or the notes of the piano, by placing one end of the stick against the instrument and the opposite one against the forehead. And this is all that can be accomplished with any of the expensive instruments sold under the name of audiphones or dentaphones.

